# 3DP Printing of Oral Solid Formulations: A Systematic Review

**DOI:** 10.3390/pharmaceutics13030358

**Published:** 2021-03-09

**Authors:** Chiara R. M. Brambilla, Ogochukwu Lilian Okafor-Muo, Hany Hassanin, Amr ElShaer

**Affiliations:** 1 Drug Discovery, Delivery and Patient Care (DDDPC) Theme, Department of Pharmacy, Pharmacy and Chemistry, School of Life Sciences, Kingston University London, Kingston Upon Thames, Surrey KT1 2EE, UK; k1907559@kingston.ac.uk (C.R.M.B.); k1738588@kingston.ac.uk (O.L.O.-M.); 2 School of Engineering, Technology and Design, The University of Canterbury Christ Church, Canterbury CT1 1QU, UK

**Keywords:** 3D printing, oral solid dosage forms, tablets, systematic review

## Abstract

Three-dimensional (3D) printing is a recent technology, which gives the possibility to manufacture personalised dosage forms and it has a broad range of applications. One of the most developed, it is the manufacture of oral solid dosage and the four 3DP techniques which have been more used for their manufacture are FDM, inkjet 3DP, SLA and SLS. This systematic review is carried out to statistically analyze the current 3DP techniques employed in manufacturing oral solid formulations and assess the recent trends of this new technology. The work has been organised into four steps, (1) screening of the articles, definition of the inclusion and exclusion criteria and classification of the articles in the two main groups (included/excluded); (2) quantification and characterisation of the included articles; (3) evaluation of the validity of data and data extraction process; (4) data analysis, discussion, and conclusion to define which technique offers the best properties to be applied in the manufacture of oral solid formulations. It has been observed that with SLS 3DP technique, all the characterisation tests required by the BP (drug content, drug dissolution profile, hardness, friability, disintegration time and uniformity of weight) have been performed in the majority of articles, except for the friability test. However, it is not possible to define which of the four 3DP techniques is the most suitable for the manufacture of oral solid formulations, because the selection is affected by different parameters, such as the type of formulation, the physical-mechanical properties to achieve. Moreover, each technique has its specific advantages and disadvantages, such as for FDM the biggest challenge is the degradation of the drug, due to high printing temperature process or for SLA is the toxicity of the carcinogenic risk of the photopolymerising material.

## 1. Introduction

Three-dimensional printing, also referred to as additive layer manufacturing, is a revolutionary, user-friendly and versatile technique that allows 3D designs to be converted into real structures. This method of manufacturing can be applied in drug delivery to fabricate 3DP drug delivery systems with precise and complex geometries through sequential layering [1,2,3,4,5]. 3D objects are designed using the CAD software, which converts the 3D model into an STL file that contains information related to the surface geometry of the 3D object. Subsequently, the STL file is sliced into layers, producing a slice file (SLI) that is then loaded into a machine (3D printer) which guides the motions of the build parts [6]. During printing, the raw material is first extruded across the x-y axis and then along the *z*-axis, to achieve the desired dimensions [7,8,9,10].

The substitution of conventional techniques with 3DP gave the possibility to provide personalised polypills to the patients, fabricated to decrease the costs of production and to improve the adherence to the therapy. Moreover, the first time that 3DP was used in the pharmaceutical area, was 1996 when a PB was employed to fabricate a 3D solid structure with a drug [10]. Subsequently, other 3D printing techniques were introduced. Indeed, in the late 1980s, was developed SLS by Carl Deckard and instead, in 1990, FDM by Sachs et al. [11,12].

3DP has a broad range of applications in different fields such as fabrication of prototypes, part consolidation, maintenance and repair in aviation and automobile industries as well as printing of human organs, prosthetics and implants in the biomedical industry. In pharmaceutical manufacturing, 3D printing can be used to accurately spread materials, allowing an easier fabrication of medications with individualised and personalised doses and polypills which contain more than one API. Moreover, 3DP allows the production of highly precise formulations, characterised by several geometries and dimensions, allowing the local drug delivery to specific organs, and offering versatile drug release rates [7].

Furthermore, 3DP technique has been used to manufacture orodispersible tablets, medical devices, doughnut-shaped tablet, polypills, channelled tablets, printed loaded with nanocapsules and duo caplets [13,14,15,16].

One of the principal applications of 3DP is the manufacturing of oral solid drug delivery systems, including a variety of complex geometries with different types of drug release profiles, such as immediate and modified as well as formulations with multiple APIs to enhance the personalisation. This is especially beneficial to patients with pharmacogenetic polymorphism or treated with drugs characterised by a narrow therapeutic index [17].

SPRITAM^®^ (levetiracetam) is the first 3DP rapidly disintegrating oral formulation, approved by FDA in 2015, produced by Aprecia Pharmaceutics. It was realised through the ZipDose technology, which is a powder bed inkjet system, that allows the production of formulations with different values of strength, showing the potential benefit of 3DP technique to produce personalised medicines answering to the specific needs of each patient. This orodispersible tablet presents a quick mouth dispersion (~11 s) and is easy to swallow [11,17].

**Figure 1:**
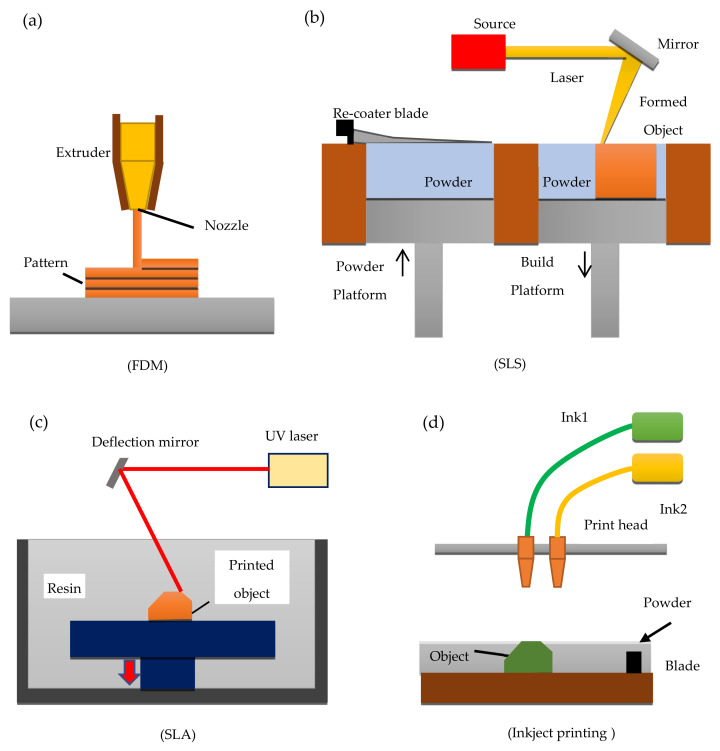
Schematic diagram of (**a**) FDM; (**b**) SLS; (**c**) SLA; (**d**) and inkjet 3DP.

3DP techniques, are based on a number of techniques to building 3D parts point-by-point, line-by-line, or layer-by-layer. Based on the manufacturing principle, the main employed 3DP techniques in the pharmaceutical area can be classified into four categories: (a) FDM; (b) SLS; (c) SLA; and inkjet 3DP. These techniques generally undergo the following steps for manufacturing; first, the 3D model is designed using the CAD software, materials are then prepared during which the defined drugs and polymers are mixed and transferred into the 3D printer. As the last step, the mix of drug and polymer is consolidated or ejected through a printer system to allow the fabrication of the 3D drug delivery system layer-by-layer [4]. Figure 1 shows a schematic diagram of FDM, SLS, SLA, and inkjet printing.

Inkjet 3DP printing was the first technique used to print 3D systems in the pharmaceutical area, where a liquid material is specifically and selectively jetted onto a substrate. The printing process is organised into three different stages, (1) droplets generation; (2) deposition of the ink droplets and interaction with a substrate; (3) solidification step. Inkjet 3DP process is known also as drop-on-powder (DoP) and it allows the formation of both a thin layer and a powder layer, where a binder liquid is deposited in a precise way. Once this binder leads to the solidification of the layer, a new powder layer is produced on the top and all the previous steps are repeated until the final formulation is manufactured [18].

Inkjet printing technology could involve a continuous jetting of droplets, where a catcher is used to collect the unwanted jetted droplets, or as with a drop-on-demand (DOD), a system where ink droplets are ejected when required. The DOD system is further classified based on the mechanism of droplets formation. The thermal inkjet mechanism involves the use of a thermal resistor that is rapidly heated to allow the vaporisation of ink, causing a vapour bubble to be formed which increases in size until a droplet is forced out of the nozzle. In piezoelectric inkjet mechanism, on the other hand, a piezoelectric material is used and once a voltage is applied to this material, this leads to the mechanical deformation, which causes the ejection of the ink droplets. Furthermore, this second mechanism is considered more advantageous than the first due to the low risk of thermal degradation of heat-sensitive ink in response to the heat [19,20,21].

The main advantages of the inkjet 3DP include a reduced number of steps to manufacture personalised tablets and control of the drug release by varying specific printing parameters such as size or surface area of the printed geometry, loading of jetted droplets, changing the droplet spacing on the substrate and also the freedom of spatial location of a drug delivery system. Furthermore, other positive aspects of this 3DP systems are the achievement of a high degree of uniformity, reproducibility, and accuracy for high-resolution applications. Moreover, inkjet printing is characterised by low costs and the possibility to deposit simultaneously, multiple materials and the amount of material released is affected by the size of the printer and by the number of jets [20].

The main challenge of the DoP process is the development of the ink itself because its physiochemical properties could strongly affect the printability. Moreover, it is relevant to develop reliable printable ink, which can also maintain its product functionality [18,19].

FDM an extrusion-based technique is the second 3DP technology analysed and is based on the deposition of different layers of molten material, which are delivered to the printer in the form of the thermoplastic polymer filaments. The polymers used for this type of 3DP system are carefully selected based on their physical properties as thermoresponsive polymers with notable biocompatibility suitable for biomedical applications. Examples include polymers such as PCL and PLGA [11].

During FDM printing, the thermoplastic material which is made available in the form of filaments is passed through a heated nozzle where the polymer undergoes a melting process to be extruded at a specific predefined rate and pressure. The melted polymer in a semi-liquid state is deposited layer by layer onto a building platform to allow them to harden [10]. The nozzle head can be moved in three different directions, leading to the deposition of a thin layer onto the platform. During the extrusion process, the material undergoes a reduction of its temperature and solidify, forming the 3D model layer-by-layer. The FDM can also present more than one print head, and each of them is controlled independently, and it allows the extrusion of several materials, whose deposition depends on the CAD model and on the printer parameters [11].

This technology has been used in the pharmaceutical field and allows the manufacturing of 3DP drug delivery systems, and both the melting temperature and the temperature of the nozzle depends on the type of polymer to be extruded [11]. It is possible to incorporate drugs into filaments using methods such as impregnation and HME. The HME process, which is the more popular method, involves using a hot melt extruder to facilitate melting, mixing and extrusion of drug, polymer and excipient. A successfully formulated and extruded mixture would emerge in the form of a drug-loaded filament.

On one hand, the main advantages of this technique include having a higher resolution compared to some of the other 3DP technologies, good mechanical strength and design flexibility that allows easy modification of some printing parameters, such as the infill percentage of printlets to obtain a desired drug release profiles of the printed dosage forms. On the other hand, the main limitation is the possible risk of drug degradation that may result from the use of a significant amount of heat. Indeed, in many cases, a temperature higher than 120 °C is used, which can lead to drug degradation, deterioration of mechanical properties, decrease of the physical stability, filaments ageing and relatively poor resolution of the 3DP objects. Moreover, the bioactivity of the drugs can be altered due to the melting temperature of the polymers to be extruded and to avoid this, it is relevant to select an appropriate drug, whose melting point is above one of the polymers [12]. By appropriately selecting the process parameters and material composition of the filament, it is possible to manufacture high-quality filaments containing the API (active pharmaceutical ingredient) [10,22]. Other drawbacks include a reduced choice of thermoplastic materials with good melting-viscosity properties for the extrusion process and the difficulty of loading thermo-sensitive drugs during the extrusion process caused by the high processing temperatures [5,11].

The third 3DP technique considered was SLA which is a simple and fast technology that has been used to create 3D objects and drug-loaded formulations through the solidification of a photoreactive liquid resin which is achieved by photopolymerisation. SLA is the first technique that produced reduced drug degradation, a property that can be considered useful for the printing of tablets with thermo-sensitive drugs [5]. SLA process uses a focused ultraviolet (UV) light or laser to selectively polymerise several layers of photosensitive and photocurable polymer materials contained inside a resin tank. Solid 3D formulations are created by the occurrence of this photopolymerisation reaction during which a liquid monomer is converted into a solid polymer. Additionally, a photoinitiator (a light-sensitive compound), which becomes active with suitable wavelengths, is added to allow the beginning of the reaction and the formation of free radicals, which will then be used to convert the liquid monomer into a solid-state [20]. Once the photoinitiator is activated at a specific wavelength, it absorbs energy to produce free radicals and begin the reaction [5,20]. SLA has the ability not only to produce large parts, but also high resolution and true 3D micro parts made from polymer or ceramic materials with accuracy better than MEMS fabrication techniques such as soft lithography [23,24,25,26,27].

Several elements can affect the energy imparted by the laser, including the power of the light, the scanning rate, the material exposed and the quantity of both polymer and photoinitiator [5,17].

The main advantages of SLA 3DP technique are versatility, the production of 3D objects with a high resolution at room temperature, the minimised heating during the printing process, absence of thermal degradation, which makes this technique more suitable to print dosage forms with thermally labile drugs included [5]. The principal drawbacks are the width of the focused layer, the reduced availability of biocompatible and biodegradable photocrosslinkable polymers, most materials are not recognised as GRAS, and the carcinogenic risk of the photopolymerising material [5,11,27,28]. Moreover, other limitations are the solubility [29], the stability of the drugs under UV light, which could be restricted and to avoid it, Kadry et al. suggested that it is relevant to carefully select the drugs and the necessity of both remove non-polymerised resin and post-curing [30].

The last technique evaluated was SLS, which is one of the most recent industry technologies used to prepare personalised solid dosage forms, either with immediate or modified drug release profiles. [18] It is a one-step, solvent-free method, where a laser beam is used to specifically bind powdered materials together and fabricate 3D structures layer-by-layer [1,31]. Awad et al. defined this technique as economical, fast, and user-friendly [9].

SLS 3DP is a solvent-free process, which not implies an alteration of the properties of the polymers, and it allows the manufacture of printlets readily dispensable, which do not need to undergo a drying process at the end of the printing [31]. Moreover, SLS is characterised by very good flexibility, which leads to the production of a broad range of dosage forms, with a huge variety of geometries and drug release profiles [9].

The principal advantages of SLS 3DP technique are its flexibility, allowing the production of a broad range of dosage forms, characterised by several shapes and drug release profiles, the sintering process, which fused drug and polymer particles, forming a strong bond between the two elements, the high resolution of the laser beam, which allows the formulation of very small and detailed units. Moreover, another relevant advantage is the high control of both the composition and the content distribution in the formulations [9]. Indeed, to fabricate these 3D structures is employed a high-resolution laser, which allows the realisation of high detailed objects, with a controllable internal structure. Moreover, this high precision also leads to a specific control of both the composition and the internal structure and thanks to these two elements, SLS is defined as an accurate and reproducible system [7,14]. Subsequently, once the printing end, the unsintered powder can be removed by airbrushing or sieving and be reused, to reduce the wastage and promote the recycling of the feedstock [14]. It can be also defined as a cost-effectiveness technology, because compared to the others, SLS resulted to be more economical.

An initial limitation of this technique was the degradation of the drugs because it was used a CO_2_ laser, which worked in the IR region of the spectra, but, nowadays, are used diode lasers with a lower intensity and no more drug degradation occurs again [1]. Furthermore, the commonly used materials are powdered forms of metal alloys, ceramics, and plastics, which need high temperatures values and high-energy lasers to be sintered. Indeed, these conditions limit the use of this technology into the pharmaceutical fields because the high-energy input of the laser can cause the degradation of the drugs if used as starting materials. Considering these limitations, SLS printing process is principally employed in the formulation of drug delivery devices, where the drug was already included or for tissue engineering scaffolds [11].

The aim of this systematic review is to identify which type of 3DP techniques is the most suitable for printing of oral solid drug delivery systems.

## 2. Materials and Methods

**Figure 2:**
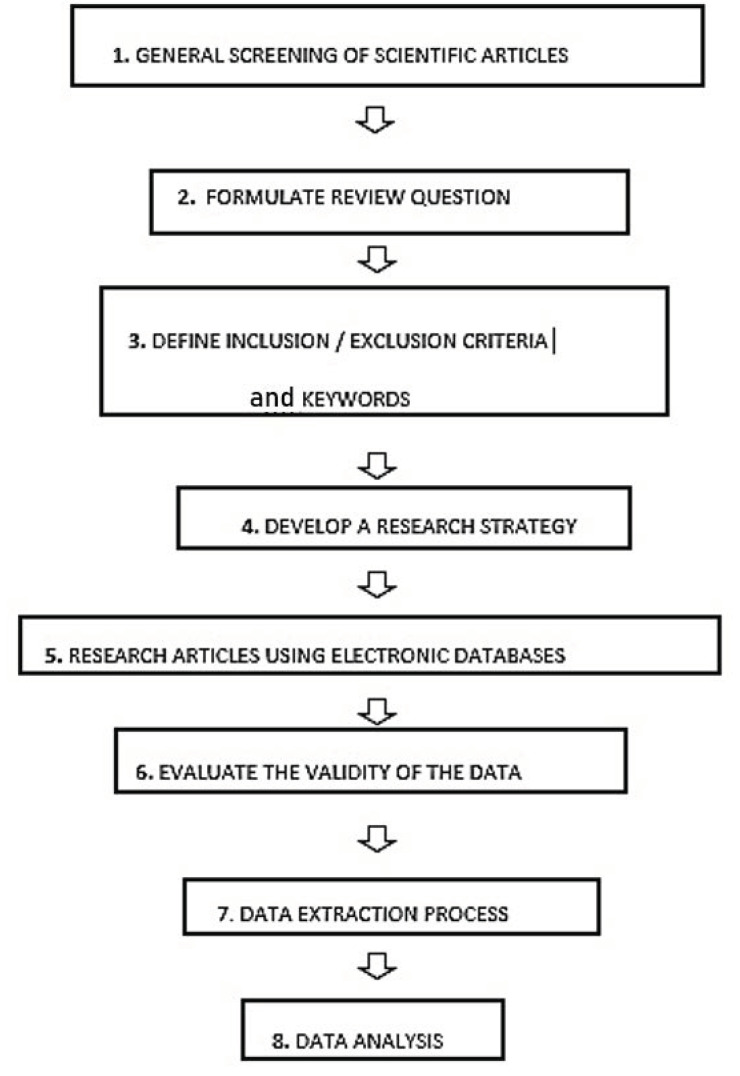
Different stages to carry out the systematic review: (1) general screening; (2) formulation of the review question; (3) definition of the inclusion and exclusion criteria and the keywords; (4) development of a research strategy; (5) research of articles using electronic databases; (6) evaluation of the validity; (7) extraction of the data; and (8) analysis of the data.

To carry out the first stage of the systematic review (Figure 2), which was the general screening of the articles, five electronic databases were used (PubMed, Google Scholar, British Library, Europepmc, Web of Knowledge). For the article screening, some specific keywords were used which can be divided into two categories. The first one is related to the different techniques which can be used in the three-dimensional printing: “three-dimensional printing”, “Additive Manufacturing”, “Fused Deposition Modeling”, “Extrusion-based 3DP”, “Selective Laser Sintering”, “Stereolithography”, “Inkjet 3DP” and their acronyms “3DP”, “AM”, “FDM”, “SLS”, “SLA”. The second group of keywords focused on the different 3DP applications; “personalised medicines”, “oral formulations”, “solid formulations”, “oral and solid drug delivery systems”, “tablet”, “capsules”. The search phrase used as input in all the electronic databases was one of the keywords reported above or their combination. In particular, the research on PubMed was conducted also considering the sections named “Similar articles” and “Cited by”.

In this systematic review, after the general screening of the articles, there was the formulation of a research question, which was “which type of 3DP technique is more suitable for printing oral and solid formulations” and the consultation of the international database “PROSPERO”, created by the University of York and funded by the NIHR (National Institute for Health Research), an online portal for the registration of any intention to carry out a systematic review. The main aim of this portal is to make the systematic review known before it is developed, to reduce their unplanned duplication through the creation of a comprehensive listing of systematic reviews. Furthermore, it also allows readers to compare different systematic reviews and highlights the several outcomes and how they could affect the results of the planned systematic review. In this study, the NIHR portal was used to determine whether similar systematic reviews have been carried out, a selection of pre-defined inclusion and exclusion criteria was outlined to determine which articles were to be accepted [32].

The pre-defined inclusion criteria were (1) 3DP techniques and (2) solid oral formulation while the exclusion criteria were (1) conventional techniques and (2) non-oral drug delivery systems. Moreover, only original research publications were included in the study, and other articles such as literature reviews, review articles, opinion articles and editorials were non-included.

During the data extraction process, there were a particular focus on some relevant aspects, which were (1) year of publication, (2) technique used, (3) formulation, (4) characterisation tests, (5) type of disease treated, (6) name of the drug and (7) aim.

## 3. Results

### 3.1. Included and Excluded Articles

**Figure 3:**
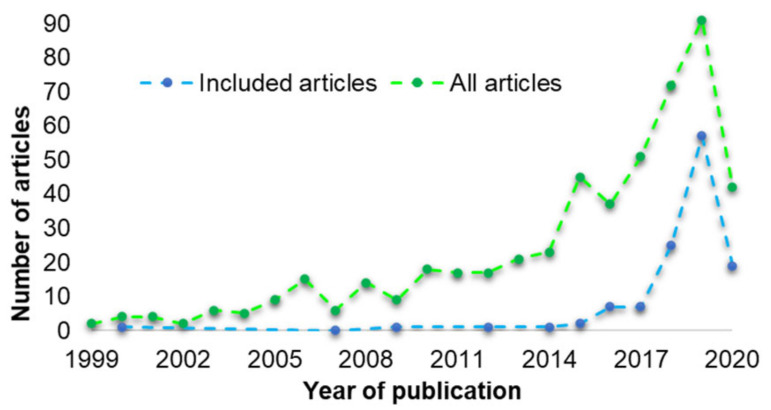
The number of 3D printed oral solid formulations published between 1999 and 2020 (green) and the number of articles included in the systematic review over the same period (blue).

The number of articles that were reported to have used 3D printing in designing oral solid formulations has increased over the years (Figure 3). In 1999, 2 articles were published. The number increased to 23 in 2014 and 91 to 2019 (green line).

**Figure 4:**
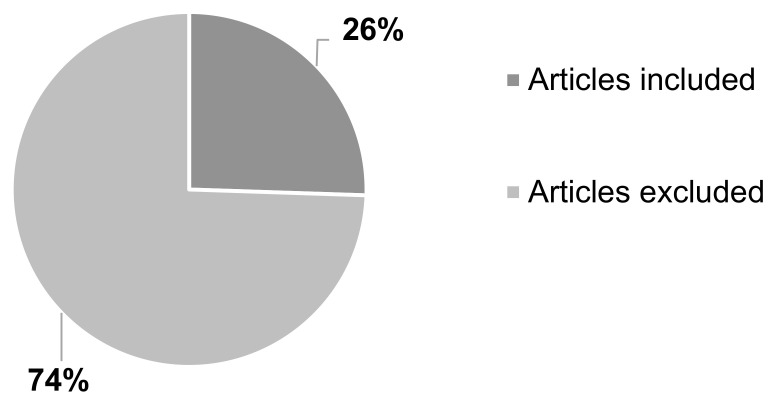
The number of articles included (dark grey) and excluded (light grey) in the systematic review, following the pre-defined inclusion and exclusion criteria.

Majority of the articles were excluded, 74% (*n* = 376) as they did not meet the inclusion criteria. Some of these articles were found to have used conventional techniques (*n* = 111), such as solvent casting, direct compression, or wet granulation. Other articles were either about non-oral formulations (*n* = 5) or duplicate studies (*n* = 18) and therefore have not been included. The number of articles included in the systematic review was 26% (*n* = 131) (Figure 4).

### 3.2. 3DP Techniques to Print Oral and Solid Formulations

**Figure 5:**
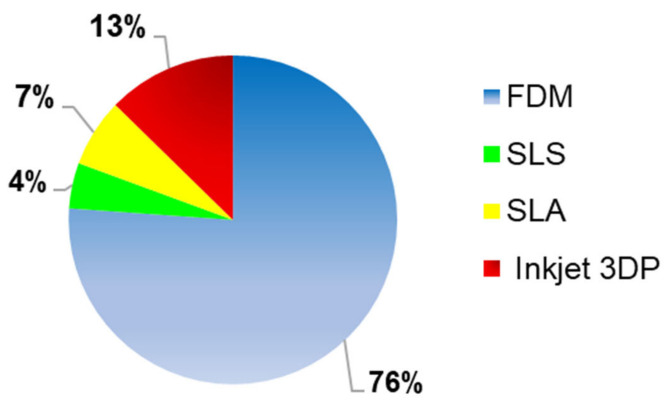
Quantification of the four 3DP techniques used in the articles included in the systematic review, (1) FDM (purple), (2) SLS (green), (3) SLA (yellow) and (4) inkjet 3DP (red).

Several 3DP techniques have been reported for the printing of oral solid formulations (Figure 5). FDM, known also as FFF, was the most used 3D printing technique, as it was applied in 76% (*n* = 102) of the studies considered for this systematic review. The second most frequently used technique was the inkjet 3DP, which was reported in 13% (*n* = 14) of the studies. Other 3DP technologies considered in the systematic review, were SLA (7%, *n* = 9) and SLS (4%, *n* = 6).

Some of the characterisation tests for tablets set by the British Pharmacopeia (BP) including drug content, uniformity of weight, disintegration, dissolution profile, friability, and hardness tests were carried out to evaluate the effectiveness of each technique in some of the studies. Characterisation test results were compared to limits and standards recommended by the BP. The acceptance value for uniformity of weight depends on the drug content while that of uncoated tablets disintegration time is within 15 min in water (authors should consider the BP general tablet monograph for coated tablets). For in vitro dissolution profile, a 70% drug release within 45 min is considered acceptable and for friability test, a weight loss of ≤1% for 10 tablets spun at 25 rpm for 100 turns (rotate 10 tablets at 50 rpm for 10 min) is recommended. Hardness is generally equal to 10–15 kg which is equal to 100–150 N. It is relevant to undergo these tests to determine if the 3DP oral and solid formulations respect the limit defined by the British Pharmacopeia [33].

Authors should discuss the results and how they can be interpreted from the perspective of previous studies and of the working hypotheses. The findings and their implications should be discussed in the broadest context possible. Future research directions may also be highlighted.

### 3.3. Inkjet 3DP

#### Characterisation Tests

In the systematic review 14 articles were included, where Inkjet 3DP printing was used to manufactured oral solid formulations, which most of them were in the form of tablets (86%, *n* = 12).

The drug content was evaluated in 93% (*n* = 13) of the included publications. For instance, Clark et al. obtained a drug content equal to 97 ± 0.4% and they reported that there was a percentage recovery, possibly due to a small amount of degradation of the API or due to an incomplete drug release from the tablets. By contrast, Cader et al. achieved a drug content of 39.8%, closed to the theoretical one of 38.4%, which was calculated considering the composition of the solid components of the formulation of the ink [19,20].

SEM analysis was carried out in 50% (*n* = 7) of the included articles. In the work conducted by Yu et al., it was analysed the inner structure of the tablets and it was observed that PVP particles did not have a regular shape, because PVP was an amorphous polymer, instead, the drug included, paracetamol was always present as a large prismatic white crystal. Moreover, the printed regions resulted were bound together through a dissolution-reprecipitation mechanism and the drug particle size was decreased, and the individual particles could not be more distinguished. Instead, in the unprinted regions, the drug particles maintained their original shapes, showing cracks and fissures [34]. Additionally, in the work done by Sen et al., the SEM analysis was performed, and it was reported an external rough structure of the tablets, with numerous porous gaps or voids on the surface [35].

The drug dissolution profile was evaluated in 93% (*n* = 13) of the articles included and 54% of the 3DP formulations showed an immediate drug release, instead 46% a sustained drug released. Clark et al. reported that 80% of the drug was released within 10 h and a complete drug released was achieved within 20 h. The main parameter which affected the drug release was the table shape. Moreover, the authors observed that a faster release was correlated to an increase of the SA/V ratio of the geometries, where the thin layer had the faster dissolution profile and the cylinder one, with the slowest one. Furthermore, this correlation between the drug release profile and the shape was also noticed by Lee et al., who also observed that the geometry of the microparticles affected several elements, such as degradation rate, stability, and drug release profile. Furthermore, the degradation of the microparticles was inversely proportional to the surface area and dimension [36]. By contrast, Sen et al. obtained an immediate drug release profile, where more than 80% of the drug was released within 30 min [29]. Considering the BP specifications, only in the work conducted by Sen et al., were met the BP limitations.

Four of the BP specifications required during the manufacturing process are the hardness, friability, disintegration time and uniformity of weight. Hardness was measured in 14% of the articles (*n* = 2) and the values were reported with different units. Sen et al. manufactured tablets with hardness values equal to 3.6–5.0 kg/cm^2^, instead, Yu et al. measured values of 63.4 ± 5.4 N/cm^2^ [28,29]. Furthermore, friability was determined in 21% of the publications (*n* = 3). In the work published by Infanger et al. the value of friability was directly proportional to the porosity of the binder particles [36]. For instance, smaller particles had values of 0.94–0.95%, which are within the BP pharmacopoeia specifications. Similar values were obtained by Sen et al., with a friability of less than 0.87% [34].

Considering the two other BP specifications, the disintegration time was measured in 29% articles (*n* = 4). Infanger et al. noticed that the disintegration time was affected by the binder viscosity. For instance, the viscosity of SL was twice than the one of SSL-FP and in the first case, the disintegration time was 1457 ± 553 s, instead the other was equal to 131 ± 71 s. This double value of viscosity led to the creation of a viscous layer before a full tablet disintegration. Moreover, this also caused long disintegration times because these newly formed gel matrices showed slow erosion, instead, the lower binder viscosity during water ingress allowed a faster absorption and a quicker disintegration [18]. Alomari et al. and Wilts et al. registered a disintegration time of 43 s and 30 s, respectively, suggesting that the manufactured tablets were classified as ODTs. Moreover, all these formulations met the BP specifications, having a disintegration time of less than 15 min [2,37].

By contrast, considering the uniformity of weight, it was evaluated in 43% (*n* = 6) of the publications. For instance, in the study conducted by Clark et al., were reported two values, one related to the batch a and the other to the batch b, which were 14.31 ± 0.04 and 14.17 ± 0.03, respectively. Furthermore, with batch a, the percentage deviation was 0.56%, whereas with batch b was equal to 0.42%. Considering the average mass, the percentage deviation, and the Pharmacopeia requirements, it was possible to conclude that both batches met the specification [20].

### 3.4. FDM (Fused Deposition Modeling)

**Figure 6:**
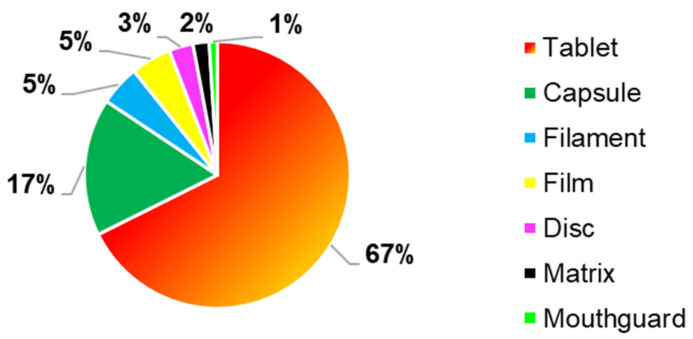
Representation and quantification of the main 3DP formulations manufactured using FDM 3DP technique, (1) tablets (red), (2) capsules (dark green), (3) filaments (blue), films (yellow), discs (pink), matrices (black) and mouthguard (light green).

Different 3DP drug delivery systems were realised using FDM as 3DP technique, (Figure 6), where tablets were the most common manufactured formulations, as it was reported in 67% (*n* = 69) of the FDM articles. Furthermore, another frequently reported formulation were capsules, which were the second most frequently 3D formulation manufactured by FDM, with a percentage of 17% (*n* = 17). Other 3DP formulations produced with this 3DP technology were filaments (5%, *n* = 5), films (5%, *n* = 5), discs (3%, *n* = 3), matrices (2%, *n* = 2) and mouthguard (1%, *n* = 1).

Tablets were more produced due to better physical-mechanical properties, such as hardness, friability, and hardness, which in most of the cases, met the British Pharmacopeia specifications.

#### 3.4.1. Characterisation Tests

**Figure 7:**
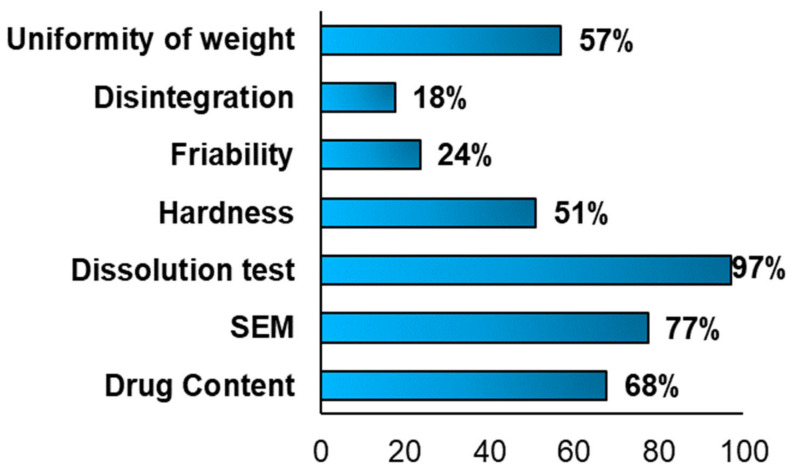
Quantification of the number (%) of FDM articles where the different characterisation tests, (1) uniformity of weight, (2) disintegration, (3) friability, (4) hardness, (5) dissolution test, (6) SEM and (7) drug content, were performed following the British Pharmacopeia specifications.

Different characterisation tests were performed to evaluate the physical-mechanical properties of the FDM 3DP formulations. (Figure 7) Moreover, each test was carried out to study specific factors and properties, such as the disintegration time, the breaking force of a tablet, the drug release profile, the 3D structure, the porosity and if during the 3D printing occurred the degradation of the drug(s).

The drug content test was carried out in 68% (*n* = 69) of the articles included in the systematic review and, in most of them, was achieved a value of drug content closed to the theoretical one, indicating an absence of drug degradation during the FDM process. For instance, in the work conducted by Genina et al., the theoretical content was 70% *w*/*w* and the actual drug content was equal to 62.2 ± 1.4% and the authors suggested that this little difference was due to the stickiness of the drug, in the form of powder, during the manufacturing process [38]. By contrast, Goyanes et al. observed that half of the drug (4-ASA) degraded at 210 °C, which was the temperature of the heated extruder. The initial drug content was 0.24% *w*/*w* and after extrusion, was equal to 0.12% *w*/*w*. 4-ASA melted and decomposed at a temperature within 130–145 °C. This suggested that it was relevant to select the appropriate drug, based on its physical properties [39].

As reported in Figure 7, SEM analysis was performed in 77% (*n* = 79) of the included articles and the results differed. For instance, Skowyra et al. observed, at the end of the extrusion process, the formation of irregular pores on the surface of the extruded filaments and voids between layers, due to the evaporation of the water content and evaporable additives. Moreover, the surface appeared irregular and rough with partially fused filaments [40]. Moreover, in a second work conducted by Goyanes et al., it was reported that the internal patterns were influenced by the infilling percentage. In fact, with a higher infilling percentage, the tablets appeared denser [41].

**Figure 8:**
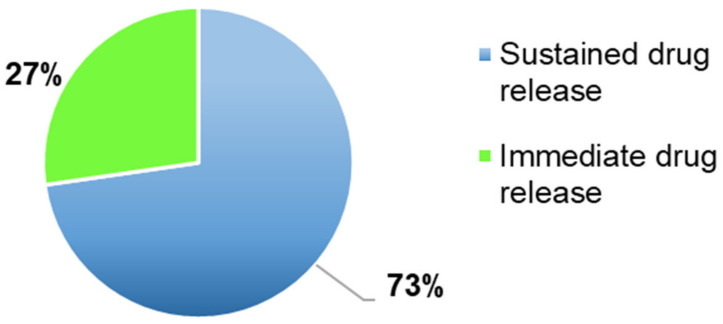
Representation of the two different drug release profiles, (1) sustained drug release (blue); (2) immediate drug release (green), which are shown by the 3DP formulations in the included FDM studies.

The most frequent characterisation test in the FDM articles included in the systematic review, was the dissolution test (Figure 7), performed in 97% of the articles (*n* = 99) to evaluate the amount of time necessary to a drug to dissolve in the dissolution media and if the drug release profile was immediate or sustained (Figure 8).

Most of the 3DP formulations presented a sustained drug release (73%, *n* = 70) had a sustained drug release profile and as defined by many authors, the drug dissolution profile can be affected by several factors. For instance, Shin et al., Tagami et al., observed that it was affected by the shape and by the size of the 3DP formulations. Moreover, smaller tablets had a quicker drug release due to a larger surface area/mass ratio, whereas larger tablets, defined also as printlets, presented a slower drug release because the SA/V ratio was smaller [42,43]. For instance, Skowrya et al. noticed that majority of the drug (>80%) was released after 12 h and over 18 h with doses equal to 4, 5, 7.5 and 10 mg. Moreover, the complete drug release was achieved within 16 h for smaller tablets and within 20–24 h for bigger ones, due to a smaller SA/V ratio [44]. The BP acceptance limit was met only by formulations with an immediate drug release profile.

Another important British Pharmacopeia specification was the hardness test, which was conducted in half of the included studies (51%, *n* = 52) (Figure 7). Moreover, this reduced evaluation could represent a limitation, being one of the specifications required by BP. In some cases, this parameter was not evaluated, because the hardness strength of the tablets was bigger than the maximum values measurable by the hardness tester (800 N), as in the work conducted by Goyanes et al. [41] and Chen et al. [45]. On one hand, the 3DP FDM formulations presented reduced values of hardness, such as, in the work performed by Khaled et al., the mesh tablets had values of 24.67 N and ring tablets of 24.72 N and In both cases, the hardness values were too small [45,46].

On the other hand, Okwuosa et al. obtained a crushing strength bigger than 350 N and Pietrzak et al. more than 490 N, indicating that the manufactured tablets presented great hardness properties, which met the British Pharmacopeia specifications [47,48]. Considering the four articles reported as an example, only the last two performed by Okwuosa et al. and Pietrzak et al. met the specifications, instead, the other two failed.

A third BP requirement was represented by the friability test, which was carried out only in 24% of the FDM included articles, possibly due to the reduction of the hardness values of the formulations. Moreover, considering the publications where this analysis was performed, all of them met the BP requirements, having a friability parameter minor or equal to one [48]. In some cases, the friability was 0%, such as in the works conducted by Okwuosa et al. and Goyanes et al. [41,49].

The fourth BP specification is the disintegration time; considering Figure 7, this test was performed in only 18% (*n* = 18) of the articles, and as reported for the friability, this can represent a limitation and an aspect that should be analysed more in future works. Taking in account the articles where the disintegration time was evaluated, only in few cases, it was possible to define the formulation as an ODT (complete disintegration underwent in less than 3 min (European Pharmacopeia) (or if less than 30 s by the FDA), such as in the works conducted by Jeong et al. and Khaled et al. [46]. In the other cases, the complete disintegration time was in a range within 5 and 15 min and in all these articles, the BP specification was met. Moreover, Palekar et al. noticed a direct correlation between the infill percentage and the disintegration time. In fact, with a higher infill percentage, more the water penetration in the formulation was reduced and faster was the disintegration time [50].

Last, but not least BP requirement to take in consideration, was the uniformity of weight, whose acceptance range depended on the average mass. For instance, if the average weight was 84 mg or less, the maximum deviation percentage was 10%, if the weight was between 84 and 250 mg, a deviation percentage of 7.5, instead of the average weight was more than 250 mg, it is accepted a deviation percentage of 5%. Analysing the above chart (Figure 7), it was reported that this test was done in 57% (*n* = 58) of the included articles and only in some of them the BP limitations were respected. For instance, on one hand, in the work conducted by Li et al., were manufactured three different tablets, with three different infilling percentage (30%, 50% and 70%) and in all the three cases, the deviation percentage was 2% and the average weight bigger than 250 mg, it was possible to define that all the formulations met the specification [50]. On the other hand, Okwuosa et al. obtained deviation percentages of 15.2% and 9.54% and has an average weight between 84 and 250 mg, both formulations did not meet the BP specifications [49]. Moreover, the same result was achieved by Goyanes et al. having a deviation percentage of 12% and an average weight more than 250 mg (309–348 mg) [51].

#### 3.4.2. Drugs Classification

**Figure 9:**
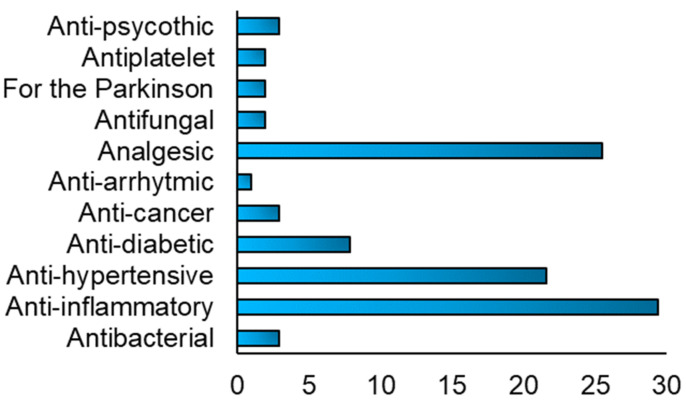
Characterization and quantification of the main type of drugs used and included in the FDM 3DP formulations, (1) anti-psychotic; (2) antiplatelet; (3) anti-Parkinson; (4) antifungal; (5) analgesic; (6) anti-arrhythmic; (7) anti-cancer; (8) anti-diabetic; (9) anti-hypertensive; (10) anti-inflammatory; and (11) antibacterial.

Different drugs were reported to be included in the FDM 3DP formulations, Figure 9, and their use depended on the type of disease to treat. Anti-inflammatory drugs, such as acyclovir and prednisolone, analgesic, as paracetamol and aspirin and anti-hypertensive, such as nifedipine and carvedilol, were the most used type of drugs used included in the FDM 3DP formulations.

As defined in the initial part, one of the main limitations of the FDM technique was the drug degradation, due to the temperature of the heated nozzle, which is correlated to the melting temperature of the polymers. As suggested by some authors, as Goyanes et al. and others, to avoid this drug degradation during the extrusion process, it was relevant to select the appropriate drug(s) and polymers to use to manufacture the 3D objects, based on their physio-chemical and mechanical properties. To allow this, we decided to evaluate the melting temperature of the polymers used in the included articles and following these values, it would be possible to define the best drug(s) to include into the drug delivery system, without any risk of drug degradation [51].

#### 3.4.3. Polymers Classification

**Figure 10:**
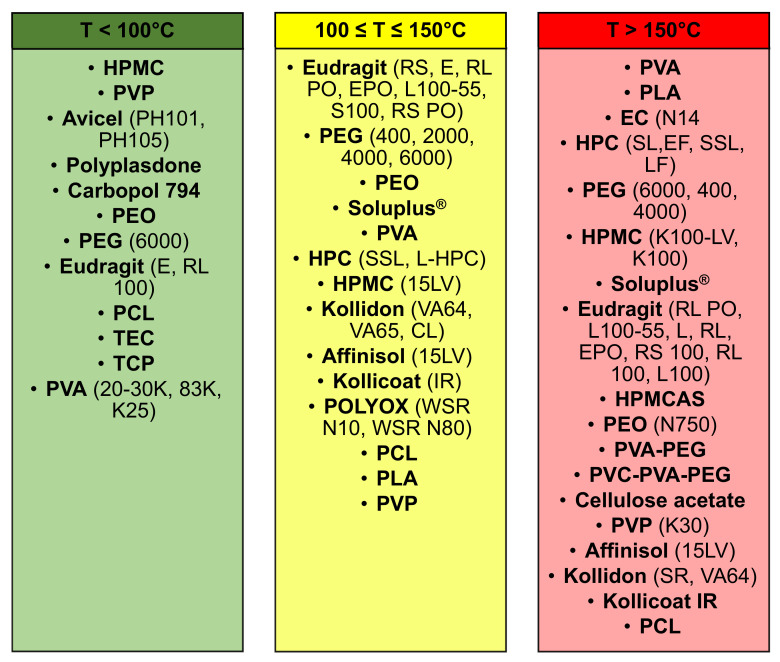
Classification of the polymers used with FDM 3DP technique based on three different printing temperature ranges: (1) T < 100 °C (green), (2) 100 °C ≤ T ≤ 150 °C (yellow) and (3) T > 150 °C (red).

In the following SmartArt (Figure 10) the polymers were classified in three main categories based on the printing temperatures reported in the included publications, (1) less than 100 °C; (2) between 100 °C and 150 °C; and (3) more than 150 °C. Moreover, the printing temperature is a relevant parameter, which needs to be defined every single time based on two parameters, the melting temperature of the polymer and the temperature at which the drug(s) will start to undergo the process of degradation.

Some polymers were reported in multiple columns because they can be extruded at different printing temperature, which was defined considering some parameters, (1) degradation temperature of the drug(s); and (2) melting temperature of the polymer(s). An example was PEG 6000, which can be printed within a range of temperature of 100–250 °C and, Considering the work conducted by Khaled et al., 3DP tablets were realised using PEG 6000 and HPMC 2910 as polymers and nifedipine and captopril were included as drugs [52]. Moreover, as printing temperature was set a value of 60 °C, whereas, considering the second column, where the printing temperature was in the range 100–150 °C, Kempin et al. used PEG 6000 with other polymers to print at 100 °C 3DP immediate-release tablets containing pantoprazole sodium sesquihydrate [53]. Instead, analysing the last column, with a printing temperature more than 150 °C, the same polymer, PEG 6000, was used by Tan et al. to manufacture sustained-release theophylline caplets. Moreover, the drug included in these caplets was theophylline and as printing temperature was defined as a value of 195 °C [54]. In all the three examples considered, the drugs did not undergo a process of drug degradation, suggesting that the defined printing temperature was appropriate and did not lead to a reduction of drug content after the printing process.

Around 19% of the included articles used temperatures below 100 °C. For instance, gastro-floating tablets were manufactured by Lin et al. using dipyridamole as drug and HPMC as polymer and the printing process was set at a temperature of 23 °C [48]. Similarly, Khaled et al. used the same printing temperature to formulate high drug loading immediate-release tablets, containing paracetamol as a drug, and as polymer was utilised PVP K25 [46].

Moreover, approximately 32% of the included publications had a printing process temperature within 100 and 150 °C. For instance, Chen et al. manufactured 3DP ellipsoid shaped gastric-floating tablets at 142 °C, using PVA as polymer and including propanol hydrochloride as the model drug [43]. In a second article, Kimura et al. realised zero-order sustained release floating tablets, using a poorly water-soluble weak base drug, itraconazole and as polymers, HPMC (hydroxypropyl cellulose) and PVP (polyvinylpyrrolidone) at a printing temperature of 135 °C [54].

By contrast, in around 52% of the FDM articles, was used a printing temperature above 150 °C to formulate oral and solid drug delivery systems. Moreover, the printing temperatures varied from 155 °C to 250 °C and there was a higher risk than the other range of temperatures, that the drug included in the formulation underwent a degradation process, due to the high extrusion process temperature. An example of this limitation was reported in the article conducted by Goyanes et al. 4-ASA and 5-ASA were used as model drugs and PVA as polymer, where only 4-ASA, whose degradation temperature was within 130–145 °C, was affected by the drug degradation, which was noticed by a reduction of the drug content from the beginning to the process (0.24% *w*/*w*) and the end (0.12% *w*/*w*). Furthermore, the authors reported that 5-ASA did not degrade because the printing temperature (210 °C) was lower than the one of its degradation point and also suggested that even if the residence time was short inside the print head, around few seconds, thermally labile drugs can undergo a significant degradation and this limitation could be overcome using a lower temperature [38]. By contrast, in other articles, such as the one conducted by Skowyra et al., extended-release patient-tailored tablets were realised setting a printing temperature of 230 °C, using PVA as polymer and prednisolone as a model drug [40], or the one performed by Jeong et al., where gastroretentive sustained-release capsules were printed at 220 °C, using PLA as polymer and baclofen as a drug, no drug degradation was observed during the printing process because the actual drug content was closed to the theoretical one [55].

### 3.5. SLA (Stereolithography)

Considering the eight included articles in the systematic review, all the 3D formulations were printlets (100%, *n* = 8). Moreover, the authors observed different structures based principally on the type of excipients.

The drug content was evaluated in 100% of the included articles (*n* = 8) and all the authors agreed that during the printing process, there was not any drug degradation. Moreover, the little difference between the drug loading value and the theoretical one was possibly due to an incomplete drug degradation from the drug-polymer matrix [5,11,30].

For instance, Wang et al. obtained a drug loading of ±5.83% *w*/*w*, which was like the theoretical one (5.9% *w*/*w*), suggesting the absence of drug degradation [5]. Same results were achieved by Martinez et al. and Kandry et al. Moreover, in the first case, the average drug content in the tablets, 3.82 ± 0.12% *w*/*w* was closed to the theoretical value of 4% *w*/*w*, which was calculated based on the formulation of the resin [10]. Furthermore, Kadry et al. had values of drug content within the range 97.18–98.75%, which respected the specified by the British Pharmacopeia. Moreover, the authors confirmed the absence of drug degradation undergoing and evaluating the UPLC spectrum [30]. Moreover, Martinez et al. evaluated the drug content inside the polypills, using HPLC and the results demonstrated that the drug loading had a range between 85–104%, which was within the acceptable range for content uniformity (85–115%) defined by the British Pharmacopeia [56].

To look at the characteristics of the printed tablets, 37.5% (*n* = 3) used SEM to evaluate the inner structure and the total porosity of the printlets and the results obtained differed based on the type of excipient(s) used. For instance, in the work conducted by Healy et al. was noticed an absence of voids on the surface of the dosage forms, which was an indication of the high level of curing. Moreover, considering the cross-sectional images of the tablets, the authors observed the absence of a homogeneous distribution of either drug and/or photoinitiator within resin, probably due to an incomplete dissolution or agglomeration process [18].

Similarly, Kandry et al. observed tablets with a smooth surface and the additive characteristics of the layer-by-layer of 3DP, where each layer had a thickness equal to 200 µm. Furthermore, to print these formulations, the authors did not use SLA, but DLP (Digital light processing), a similar technique, which differed for the light source to cure the resin. Indeed, the SLA uses lasers combined to galvanometers, instead DLP, the light source was represented by a digital light projector screen. Kandry et al. defined this 3DP technique as having a superior capability to print tablets, with uniform weights and dimensions [30].

By contrast, Krkobabić et al. noticed a variation of the internal structure of the printlets based on the presence or absence of excipients. For instance, when PEG 400 was used, the authors observed a crack propagation during the dissolution test, which had a role in the printlets erosion. Moreover, another example was represented by mannitol, which caused the formation of an irregular internal structure. Indeed, considering the cross-section of the tablets after the dissolution test, it was possible to see cracks, which indicated that tablet capping under elevate pressure, determined by the osmotic effect of mannitol [57].

Another important test that was conducted to evaluate the effectiveness of SLA technique, was the drug dissolution test, which was performed in 100% (*n* = 8) of the articles. In particular, the authors evaluated the correlation between several parameters, such as the ratio between cross-linkable polymers in the tablets, the structure of the tablets, their geometry, and the addition of excipients on the drug release rate. The authors concluded that it was relevant to select the suitable amount of photocrosslinkable polymer to manipulate the drug release rate and that both the geometry of the tablets and the addition of excipients can affect the dissolution profile.

Wang et al. evaluated how the ratio between the cross-linkable polymers in the tablets, PEDGA/PEG 300 could affect the dissolution profile. On one hand, they observed an indirect correlation between the amount of PEDGA and the drug release rate. For instance, when the amount of PEDGA was equal to 35%, 100% of paracetamol was released after 10 h, whereas, when it was 65% and 90%, the quantity of paracetamol released was 84% and 76%, respectively. On the other hand, the correlation between the amount of PEG 300 and the drug release rate was direct, indicating that with an increase of the first, there would be an increase of the second [5]. Moreover, in an experiment conducted by Heavy et al., it was also highlighted the relation between cross-linkable polymers and drug release rate. Additionally, these authors, as did Wang et al., concluded that it was relevant to select the suitable photocrosslinkable polymer to manipulate the drug release, because, on one hand, if the amount of polymer were too low, there could be surface cure problems. On the other hand, if it was too high, this concentration could reduce the amount of UV light that would penetrate through the lower layers, determining inadequate curing. Moreover, the formulations were characterised by a sustained drug release rate profile over the 24 h [18].

Similarly, Martinez et al. analysed the effect of the geometry of the printlets and the addition of excipients on the dissolution profile, realising three types of printlets. In the case of type I, a cylindrical printlet, it did not reach a complete drug release rate after 20 h, but it was within a range of 22–80%. Instead, type II, a ring-shape tablet, presented a faster drug release, because there was an increase of the surface area. Furthermore, the third type of polypill was realised to evaluate how a soluble filler (solubilising agent), PEG 300, can modify the dissolution. Indeed, the authors observed that its addition, led to an improvement of the drug release rate, compared to the type I, and the increase was affected by the type of drug. For instance, with paracetamol and aspirin, it was reached a complete drug release in 20 h, instead, with prednisolone, a poorly soluble drug, was increased to 45%, but it was never achieved a complete drug release after 20 h [56].

The hardness, defined also as breaking force, is an indicator of the mechanical properties of the tablets, was evaluated in 37.5% of the studies (*n* = 3) and one of these articles, the authors noticed that the hardness and the tensile strength were affected by the number of excipients, such as PEG 400, PEDGA, water and mannitol.

In the study conducted by Madzarevic et al., eleven different formulations were manufactured, with a different composition (% *w*/*w*) in terms of the amount of PEGDA, PEG 400, Water, riboflavin, and ibuprofen. For every single formulation was calculated the hardness values and based on their hardness values, the formulations can be classified into two categories, (1) met the British Pharmacopeia requirement, having a hardness value between 100 and 150 N, instead (2) did not meet the BP requirements, because the values were lower than 100 N. Seven of the eleven formulations did not meet the specifications, because their hardness values were between 19.00 ± 8.66 N and 47.33 ± 3.21 N. Instead, formulation number 3, 4, 6 met the BP specifications, having a hardness value within the range of 92.33 ± 29.02 N and 132.33 ± 18.88 N, whereas with formulation number 7 the hardness was not determined [28].

Another characterisation test required by the British Pharmacopeia was the uniformity of weight, which was evaluated in 50% (*n* = 4) of the studies. In the work conducted by Heavy et al., the weight uniformity of all the printlets had a range between 81.7–118%, with a percentage deviation of 36.3% and, considering that the average mass was 1621 mg and the prescribed limit of weight varies according to British Pharmacopeia, these values did not respect the prescribed limits [19].

Similarly, in the publication carried out by Kadry et al., the weight of the tablets was between 133.70 mg and 174.23 mg. The average weight was 154.0 mg and the range was 86.8–113%, with a difference of 26.2%. Considering the prescribed limits of weight variation set by the British Pharmacopeia, these tablets did not meet the Pharmacopeia Specifications [30]. Both formulations did not meet the Pharmacopeia requirements, having a percentage of deviation bigger than the one defined by the British Pharmacopeia.

### 3.6. SLS

To carry out the systematic review were considered 6 articles (4%) of the included publications. The drug content, determined by HPLC analysis, was evaluated in the major part of the included publications, 83% (*n* = 5), and, in all these articles, the authors reported and agreed that no drug degradation, defined also as drug loss, occurred during the SLS printing process because the drug(s) content was close to the theoretical value [1,7,12,15,31]. For example, Allahham et al. quantified the drug loading of the printlets and they obtained that it was very close to the theoretical values. In the case of formulation one, the drug loading value was equal to 98.6% ± 2.2, instead, with formulation two was to 98.1% ± 1.7 [1].

Similarly, in the study conducted by Fina et al., it was demonstrated that no drug(s) degradation occurred during the SLS printing process and to confirm it, the drug content was determined and in all the cases, the values were closed to the theoretical drug loading (5, 20 and 35%). Moreover, another element to highlight their theory was considering the HPLC spectrum and if no other peaks than the ones of the drug were observed, this indicated that no drug degradation occurred. This is what the authors noticed during their work [11].

SEM analysis was performed in 83% (*n* = 5) of the included articles and the authors noticed an indirect correlation between two parameters, the laser scanning speed and the total porosity of the tablets, a correlation between the structure of the printlet and the intensity of the sintering process and between the structure of the printlet and the type of excipient added, such as mannitol.

Fina et al. observed the indirect correlation between the laser scanning speed and the porosity values of the printlets. Indeed, with an increase of the speed from 100 to 300 mm/s, there was a decrease of the sintering of powder particles, and this led to an increase of the overall porosity, which, respectively, determined an improvement of the disintegration and dissolution of the printlets [31].

On contrast, Awad et al. noticed the correlation between the structure of the printlet and the intensity of the sintering process. The single miniprintlets underwent a more intense sintering process, whereas the dual miniprintlets, had a low-intensity sintering process, which led to the creation of a higher space volume within the particles [5]. In another work, Fina et al. highlighted the influence of different formulations on the total porosity values. For instance, Kollicoat formulations had similar porosity values, instead, Eudragit dosage forms presented a reduction of the total porosity, directly proportional to the increase of the drug content [12].

The drug dissolution profile was evaluated in all 100% (*n* = 6) of the included articles. The authors reported that the drug release rate was affected by several factors, such as formulation, laser scanning speed, the structure of the tablet (single miniprintlets or dual miniprintlets), shape and open porosity [31].

Fina et al. demonstrated that the drug release rate was influenced by the laser scanning speed. Indeed, three different laser scanning speeds were tested and the correlation between the laser scanning speed and the drug release rate depended on the formulation. For instance, HPMC formulations showed a decrease of the dissolution rate with an increase of the laser scanning speed (100 mm/s = 4 h, 300 mm/s = 2 h). Whereas Kollidon formulations presented a direct correlation between drug release rate and laser scanning speed (100 mm/s = 60 min, 200 and 300 mm/s = 10 min). Moreover, both formulations had an immediate drug release profile [31].

Furthermore, both Awad et al. and Goyanes et al., in their respective studies, noticed that the drug dissolution profiles were influenced by the structure of the tablets. On one hand, Awad et al. observed that single miniprintlets shows a sustained-drug release, where after 24 h about 71% was released, instead, the dual miniprintlets had an immediate drug release profile, undergoing a complete drug release in 30 min. On the other hand, Goyanes et al. formulated two different types of printlets, cylindrical and lattice. In both cases, 4 excipients were used and based on the type of printlet, they showed a different drug release, sustained for the cylindrical ones (for instance, PEO formulation released 60% of the drug in the first 2 h and the rest, 40%, in the following 4–5 h) and immediate for the others (such as PEO underwent a complete drug released in 10 min) [15].

Similarly, Fina et al. noticed the correlation between the drug release rate and both drug content and porosity values. Indeed, Kollicoat formulations, with an immediate drug release profiles, had an improvement of the drug released based on an increase of the drug content. For instance, K5 took 5 h to dissolve completely, whereas K35 approximately 5 h. On the other hand, the drug release profile of Eudragit formulations was influenced by both the drug content and the porosity values. Indeed, in 2 h, E5, with the highest porosity values, released 14% of the drug, instead of E35, with a reduced porosity, released only 6% of the drug [21].

The hardness of the printlets was evaluated in 83% of the articles (*n* = 5). Some authors highlighted the indirect correlation within the laser scanning speed and the breaking force values, such as Fina et al. In the articles included, more than half of the manufactured tablets were characterised by a reduced hardness and this can represent a limitation.

Fina et al. noticed the correlation between the laser scanning speed and the breaking force values. For instance, 3DP printlets manufactured at higher laser scanning speed had a value equal to 14 N (weak), instead, the ones realised at a lower speed, presented a breaking force value of 171 N (strong) [26]. Moreover, Allahham et al. manufactured two different formulations of printlets, which differed for the percentage of excipients and they both showed similar breaking force values, 14.7 N and 18.5 N. Moreover, the authors reported that these dosage forms did not break readily during the manipulation process and due to this, they presented appropriate properties for handling [1].

By contrast, in the work conducted by Goyanes et al., the three cylindrical printlets were strong, having a breaking force value of 280 N. On one hand, the lattice printlets of EUD L and EC were more friable, having a breaking force value of 15 N. On the other hand, the lattice tablets of EUD RL broke into small pieces during the test and due to this reason, it was impossible to detect their breaking force values. The breaking force values depended on both the type of printlets (cylindrical and lattice) and the typology of excipient (PEO, EUD L, EC or EUD RL) [39]. Moreover, in this experiment, the manufactured printlets were characterised by a high hardness.

The friability values were calculated in only 17% (*n* = 1) of the included studies and Fina et al. registered values less than 1% (range values 0.02–0.53%), which met the BP requirements for uncoated tablets, making them suitable for handling and packing [12].

Another relevant test carried out to evaluate the printlets properties was the disintegration test, which was done in 67% (*n* = 4) of the included studies. In the work conducted by Fina et al., three formulations were manufactured, and the authors noticed that with an increase of the laser scanning speed was correlated to a decrease of the disintegration time. For instance, the formulation realised at 100 mm/s, showed a disintegration time of over 600 s, instead, the two manufactured at 200 and 300 mm/s, underwent a complete disintegration in 320 s and 4 s, respectively. Based on the definition of ODTs given by the European Pharmacopeia and FDA, only this last formulation (K300), can be defined as ODT. The authors suggested that this reduction was correlated to the less energetic sintering process. Subsequently, this led the powder particles to get in contact with the dissolution medium and, the improved porosity values determined a reduction of the disintegration time [31].

Moreover, other authors who produced ODTs formulations were Allahham et al. and they calculated that the printlets disintegration times of the printlets (15 s) were in line with the values of the commercial formulation (14.3 ± 2.7 s) [1]. Similar values were achieved by Awad et al., through the fabrication of printlets without any pattern or with Braille A and Q, and they obtained values equal to 4.0 ± 1.3 s for printlets without any pattern, 4.3 ± 1.5 s with Braille A and 5.2 ± 1.2 s for Braille Q [58].

By contrast, the only authors who did not manufacture ODTs, were Goyanes et al. were reported the disintegration values of only two printlets, PEO and EUD RL with a lattice structure. PEO showed a complete disintegration in 10 min, instead EUD RL within 120 min. Following the definition of ODTs given by the European Pharmacopeia and FDA, neither of them could be considered an oral disintegration tablet [15].

The weight uniformity was evaluated in 67% (*n* = 4) of the included articles. The authors noticed that it was influenced by several variables, such as the laser scanning speed and the types of formulations. It was observed an indirect correlation within the laser scanning speed and weight uniformity.

Moreover, Fina et al. highlighted an indirect correlation between the laser scanning speed and the weight uniformity. Indeed, an increase of the laser scanning speed led to an arise of the number of necks inside each layer, to a reduction of the empty spaces and the formation of more spaces for powder particles to be sintered and this determined the manufacture of denser and heavier printlets [31].

Furthermore, in the work conducted by Awad et al. to produce 3D printlets with Braille and Moon patterns, the average weight of Braille printlets was 171.3 mg, with a range from 164.1 ± 1.6 mg (printlets with a one Braille dot) to 178.1 ± 5.6 mg (average weight printlets with 5 Braille Dots). The authors noticed an increase in the average weight of 3.8% with the addition of one Braille Dot. By contrast, the Moon printlets showed an average weight of 165.8 mg, with a weight range within 162 ± 1.7 mg (weight with letter H) to 171.1 ± 5.9 mg (average weight printlets with letter N) [58].

On one hand, the Braille patterns with a one Braille date, the uniformity of weight presented a range of 99–101%, with a percentage of deviation equal to 2%. Considering the Pharmacopeia requirements, it was possible to conclude that these tablets met them. On the other hand, the 5 Braille dots had a range equal to 96.9–103%, with a percentage of deviation of 6.1%. Considering the average mass and the Pharmacopeia requirements, it is possible to conclude that both tablets met the specification. Instead, considering the Moon patterns, the printlets with letter H had a percentage deviation of 2% (99–101%), whereas the ones with letter N, had a value equal to 6.4% (96.6–103%). Additionally, these printlets met the BP specification.

## 4. Discussion

This systematic review was carried out to determine which 3DP technique resulted as the most suitable to formulate oral solid drug delivery systems. As the first step, the articles were screened and 514 were selected based on both the keywords and on the review question. Secondly, the publications were classified into two groups, included, or excluded, following the pre-defined inclusion and exclusion criteria. Based on this classification system, 26% articles (*n* = 131) were included and 76% (*n* = 376) were excluded (Figure 4).

The initial study was focused on quantifying which of the several 3DP techniques evaluated, occurred more often in the included publications and as reported in Figure 5, FDM, with a value of 76% (*n* = 102), was the 3DP technique most used to formulate oral solid drug delivery systems. Moreover, the main advantages of this technology were a high resolution, good mechanical strength, and the possibility to manufacture 3D formulations with a specific drug release profile, obtained varying some printing parameters. By contrast, as reported in the data analysis chapter, one the biggest challenges of FDM technique was the degradation of the drug, due to the selection of a printing temperature above the degradation point of the drug, causing deterioration of its mechanical properties and reduction of the drug content. Furthermore, considering how relevant was this parameter in the FDM printing process, the polymers, as reported in Figure 9, were classified into three different categories, (1) T < 100 °C, (2) 100 °C ≤ T ≤ 150 °C, (3) T > 150 °C. The third category represented the one where the degradation of the drug(s) included in the 3DP formulations could occur more often, due to the use of a high printing process, defined also as extrusion temperature of the nozzle in the print head. The degradation of the drug is a relevant drawback of FDM because it does not allow the inclusion of thermo-sensible drugs inside the filaments, such as 4-ASA.

Each technique has its specific advantages and drawbacks. For instance, one of the biggest challenges of SLA is the cancerogenic risk of the photopolymerising material. Moreover, in no one of the included SLA studies was performed a test to evaluated the toxicity of the photopolymerising material and this non-evaluation represents a huge drawback and a reason for which this technique cannot be defined as the most suitable to print 3DP drug delivery systems. Moreover, as a future perspective, it is important to carry out this test, to evaluate the safety of the 3DP formulations before being administered to the patients.

The characterisation tests were performed and evaluated, to study the 3DP drug delivery systems, quantifying in how many articles were performed and which were the main factors affecting the results. During the development of the systematic review, six different characterisation tests (Table 1) were performed, to evaluate the quality and the mechanical properties of the 3DP oral solid formulations, following the BP specifications. In the following table, it is possible to observe that each test was performed with a different percentage with the four 3DP techniques.

The drug content was evaluated in most of all the techniques analysed, with a percentage above 90%, whereas only with FDM technique the value was a little lower compared to the others (68%). This could represent a factor to improve in the future publications, where the FDM 3DP is used as printing technology, because as noticed early, once of the biggest challenges of this system, was the degradation of the drug(s), due to the high printing temperature, causing a reduction of the drug content in the 3DP formulation. As shown in Figure 7, several polymers were used to manufacture 3DP formulations, and each of them has its specific degradation point and different applications. Furthermore, the choice of the printing temperature process depends on the degradation point of the drug and the melting temperature of the polymer. For instance, 4-ASA has a degradation temperature around 130–145 °C and to avoid the degradation, it is important to select both a printing temperature process below this range and a polymer, whose melting point is below the degradation temperature of the drug.

The drug dissolution test was performed to evaluate the type of drug release profile, if immediate or sustained and the amount of drug released in a specific amount of time. It was carried out in almost all the articles included, for all the four techniques evaluated, with a percentage value equal to 100%. As represented in Figure 8, 76% of the 3DP formulations had a sustained drug released, instead 24% an immediate one and as shown in Figure 6, the main FDM 3DP formulations were tablets (67%). Moreover, tablets are the preferred 3DP formulations, compared to the others, due to better physical and mechanical properties, such as hardness and friability. Some authors noticed that some parameters affected the drug release profile, such as the tablet shape and the surface area to volume ratio (SA/V). Referred to this last parameter, it was observed an inverse correlation between the size and the drug release profile. Indeed, with a decrease in the size, there was an increase in the SA/V ratio and improvement of the drug release profile [20,41,42]. By contrast, other authors noticed that the drug release profile was affected by the amount of photocrosslinkable polymers, excipients, laser scanning speed, the intensity of the sintering process and porosity values [5,7,11,30]. Furthermore, a relevant consideration to carry out, it is that the factors which can affect the drug release profile, were different, based on the 3DP technique evaluated.

The hardness test defined also as breaking force test was most performed in the SLS included articles, with a percentage equal to 83%. Considering FDM 3DP technique, this test was performed in only half of the cases (51%), whereas with inkjet 3DP in only a reduced number of publications [20] and some cases the hardness could not be evaluated, because the value was above the maximum limit measurable by the hardness tester (>800 N), whereas in the works conducted by Khaled et al. the hardness had values within a range of 24.67 N to 24.78 N [40,44], instead of in the article carried out by Okwuosa et al. and Pietrzak et al. was 350 N and 490 N, respectively [46,47]. The main parameters which affected the hardness were the number of excipients added in the formulation and the laser scanning speed [1]. For instance, Fina et al. noticed an inverse correlation between the breaking force values and the laser scanning speed [25]. Moreover, considering how relevant is this parameter, it is important in the future works, to carry out this test, to evaluate if the 3DP drug delivery system is suitable or not for the intended use and if it meets the BP specifications.

The friability test was performed in a limited number of publications (17–24%) in three of the four 3DP techniques, whereas with SLA was not performed. A possible explanation could be due to the limited hardness values of the 3DP formulations, which were not able to undergo this type of test and this reduced friability evaluation represents an element that needs to be improved in the future works, being one of the tests required by the British Pharmacopeia (BP).

Furthermore, the main parameter which affected the friability was the porosity of the binder particles, which had a direct correlation with the friability values, as reported by Infanger et al. [18]. Similarly, with all the FDM 3DP formulations the BP requirements were met, having friability values ≤1%.

As for the friability, the disintegration test was not performed with SLA 3DP technique, whereas, with SLS was evaluated in 67% of the included articles. Moreover, this test also allowed to define if an immediate drug release profile formulation can be defined as ODT, based on the European Pharmacopeia and FDA definitions. For instance, in the work conducted by Jeong et al. and Khaled et al., the formulations had a disintegration time of less than 3 min and can be classified as ODTs [45,54]. Furthermore, the main parameters which affected the disintegration time were the binder viscosity [18], the infill percentage, water content and the printing pattern. Indeed, Palekar et al. observed a direct correlation between the infill percentage and the disintegration time. Moreover, higher is the infill percentage, less water can penetrate inside the formulation and faster will be the disintegration time [49].

The uniformity of weight was prevalently evaluated with SLS, 67% and as for the other characterisation tests, some parameters were affecting the weight uniformity, such as the laser scanning speed. For instance, Fina et al. noticed an inverse correlation within the uniformity of weight and the laser scanning speed. Indeed, an improvement of the laser scanning speed, led to an increase of the number of necks in each layer, to a subsequent reduction of the empty spaces and to the creation of more spaces, which can be occupied by the powder particles to be sintered. Consequently, all these elements allowed the production of denser and heavier tablets [30].

## 5. Conclusions and Future Perspectives

3D printing is a revolutionary technique, which gives the possibility to manufacture patient-personalised formulations, offering a decrease of the costs and an increase of the adherence to the treatment. Moreover, one of its main application is the formulation of oral solid drug delivery systems and the four 3DP techniques, which have been more used for their manufacture are FDM (76%), inkjet 3DP (13%) SLA (7%) and SLS (4%).

Considering Table 1, all the observations made related to the six characterisation tests and how frequently they have been evaluated, it is possible to conclude that SLS 3DP has been the technique where most of them have been evaluated with the highest percentages, except for the friability test, which was performed in only 17% (*n* = 1) of the included publications.

However, it is not possible to define which is the most suitable technique to print oral solid formulations, because every 3DP technique has its advantages and disadvantages and the selection of the specific printing technique depends on the type of formulation which is desired to manufacture and on the physical-mechanical properties to achieve, such as hardness, friability or drug dissolution profile. Moreover, some improvements should be carried with all the four techniques evaluated, to perform all the six characterisation tests and meet the acceptance criteria defined by the BP specifications for the manufacture of tablets.

The 3DP of medication is a new field in its infancy facing a number of challenges. One obstacle is the suitability of the 3DP techniques, as a number of the techniques could deter the stability of thermolabile drugs. Additionally, it is still challenging to prepare tablets that meet the legal requirements of the regulatory bodies and pharmacopeia. Another issue is the limited availability of ink materials that are compatible and suitable for preparation of oral medications.

## Figures and Tables

**Table 1:** Schematic summaries of the six characterisation tests (drug content; dissolution profile; hardness; friability; disintegration; and uniformity of weight) performed with the four 3DP techniques (FDM; SLS, SLA; inkjet 3DP) and quantification in how many articles were evaluated.

	Drug Content	Dissolution Profile	Hardness	Friability	Disintegration	Uniformity of Weight
FDM	68%	97%	51%	24%	18%	57%
SLS	83%	100%	83%	17%	67%	67%
SLA	100%	100%	37.5%	0%	0%	50%
Inkjet	93%	93%	14%	21%	29%	43%

## Abbreviations

3DP	Three-dimensional printing;
3D	Three-dimensional;
CAD	computer-aided design;
PB	powder bed 3D printer;
SLS	selective laser sintering;
FDM	Fused Deposition Modelling;
API	active pharmaceutical ingredient;
FDA	Food and Drug Administration;
SLA	stereolithography;
PRISMA	Preferred Reporting Items for Systematic reviews and Meta-analysis;
PROSPERO	International Prospective Register of Ongoing Systematic Reviews;
NIHR	National Institute for Health Research;
AM	Additive Manufacturing;
BP	British Pharmacopeia;
DoP	drop-on-powder;
DOD	drop-on-demand;
SEM	Scanning electron microscopy;
PVP	polyvinylpyrrolidone;
SA/V	surface area to volume ratio;
ODT	orally disintegrating tablet;
PCL	polycaprolactone;
PLGA	poly(lactic-co-glycolic acid);
HME	Hot melt extrusion;
HPMC	hydroxypropyl methylcellulose;
PEO	polyethylene oxide;
PEG	polyethylene glycol;
TEC	triethyl citrate;
TCP	tri-calcium phosphate;
PVA	poly(vinyl alcohol);
HCP	hydroxypropyl cellulose;
EC	ethyl cellulose;
HPMCAS	hydroxypropyl methylcellulose acetate succinate;
4-ASA	4-Aminosalicylic acid;
5-ASA	5-Aminosalicylic acid;
UV	ultraviolet;
GRAS	generally recognised as safe;
UPLC	ultra-performance liquid chromatography;
HPLC	High Performance Liquid Chromatography;
DLP	Digital light processing;
PEDGA	poly(ethylene glycol) diacrylate;
USP	United States Pharmacopeia;
CO_2_	carbon dioxide;
IR	infrared;
SD	standard deviation;
EUD	Eudragit;
FFF	Fused filament fabrication;
STL	standard tessellation language;
SLI	slice file;
TGA	thermogravimetric analysis;
ODPs	orodispersible printlets;
TPO	diphenyl(2,4,6-trimethylbenzoyl) phosphine oxide;
PET	poly(ethylene terephthalate);
FD-DDDs	fast-disintegrating drug delivery devices;
MET	metformin hydrochloride;
Para	Paracetamol;
Asp	Aspirin.
